# Constructing and exploring neuroimaging projects: a survey from clinical practice to scientific research

**DOI:** 10.1186/s13244-024-01848-9

**Published:** 2024-11-15

**Authors:** Ziyan Chen, Abraham Ayodeji Adegboro, Lan Gu, Xuejun Li

**Affiliations:** 1 Department of Neurosurgery, Xiangya Hospital, Central South University, Changsha, China; 2 Hunan International Scientific and Technological Cooperation Base of Brain Tumor Research, Xiangya Hospital, Central South University, Changsha, China; 3 School of Foreign Languages, Central South University, Changsha, China; 4 Xiangya School of Medicine, Central South University, Changsha, China

**Keywords:** Neuroimaging, MRI, Data management, Ethics

## Abstract

**Abstract:**

Over the past decades, numerous large-scale neuroimaging projects that involved the collection and release of multimodal data have been conducted globally. Distinguished initiatives such as the Human Connectome Project, UK Biobank, and Alzheimer’s Disease Neuroimaging Initiative, among others, stand as remarkable international collaborations that have significantly advanced our understanding of the brain. With the advancement of big data technology, changes in healthcare models, and continuous development in biomedical research, various types of large-scale projects are being established and promoted worldwide. For project leaders, there is a need to refer to common principles in project construction and management. Users must also adhere strictly to rules and guidelines, ensuring data safety and privacy protection. Organizations must maintain data integrity, protect individual privacy, and foster stakeholders’ trust. Regular updates to legislation and policies are necessary to keep pace with evolving technologies and emerging data-related challenges.

**Critical relevance statement:**

By reviewing global large-scale neuroimaging projects, we have summarized the standards and norms for establishing and utilizing their data, and provided suggestions and opinions on some ethical issues, aiming to promote higher-quality neuroimaging data development.

**Key Points:**

- Global neuroimaging projects are increasingly advancing but still face challenges.
- Constructing and utilizing neuroimaging projects should follow set rules and guidelines.
- Effective data management and governance should be developed to support neuroimaging projects.

**Graphical Abstract:**

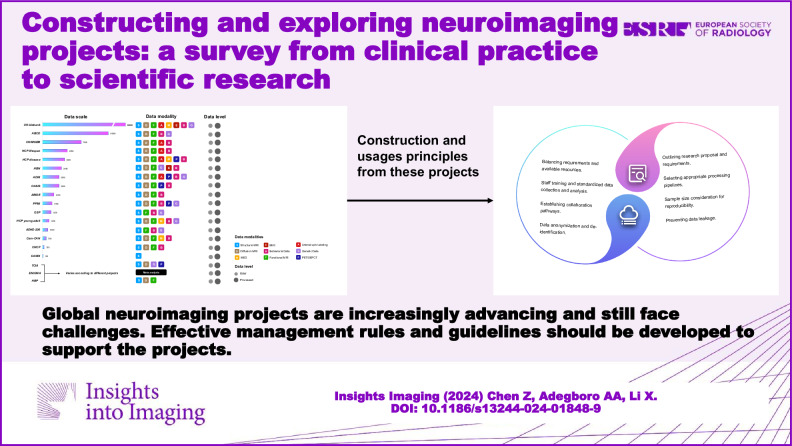

## Introduction

During the past decades, huge amounts of neuroimaging data from different populations and individuals with various physiological and pathological states, including magnetic resonance imaging (MRI), electroencephalography (EEG), magnetoencephalography (MEG), and other modalities, have been continuously collected and utilized in clinical and research fields. MRI especially, is non-invasive with multiple modalities that enable the acquisition of comprehensive information about the whole brain, both at structural and functional levels. This versatility makes MRI an invaluable resource for clinical practice, and as a result, an increasing number of studies and projects are utilizing multimodal MRI for their research [1].

Various large-scale research projects have been conducted with many data sets released, aiming to interpret the changes in brain structures and functions during human or other organisms’ development and disease states from a neuroimaging perspective. Other information, such as genetic data, behavioral data, clinical laboratory examinations, and various scale data, were also collected in different research projects to provide a comprehensive understanding of physiological development and disease progression.

Moreover, recent advancements in artificial intelligence (AI) models, fueled with diverse data, have improved computational performance and speed [2]. Leveraging diverse AI, large-scale data sets, and various data types is not only enhancing our understanding of human and non-human nervous systems but also opening avenues for disease treatments [3, 4]. Simultaneously, the analysis of the brain signal transmission and processing, information filtering, along mathematical abstraction has also contributed to the evolution of AI, making it increasingly closer to the human brain in terms of thinking patterns and signal processing.

However, some concerns exist with the generation of vast amounts of data. For example, the imbalanced development of large neuroimaging projects across the world, and institutions and researchers’ difficulties in conducting large-scale research surveys targeting large populations [5, 6]. Also, new challenges are arising in data management, including issues related to anonymization, privacy protection, and ethical considerations [7, 8].

Thus, we begin our review by listing excellent neuroimaging projects globally; which represent significant international collaborations and have contributed immensely to our understanding of the brain. Drawing on the experience from these, we then discuss the benefits and risks associated with conducting and establishing large-scale data sets, as well as considerations related to data sharing and population privacy protection [9–11]. Furthermore, from the perspective of researchers in clinical settings, we explore efforts that can be made to enhance collaborations across the developmental stages and between research centers, promoting advancements in the field.

## An overview of neuroimaging projects

Large-scale neuroimaging projects have been undergoing a rapid increase and development during the last 20 years. Since 2004, a collaborative effort has been underway to advance the mapping of human brain structural and functional connections and improve medical care for brain-related diseases. This initiative has brought together various units and centers, pooling their expertise and resources to accelerate progress in the field.

The National Institutes of Health (NIH) Blueprint for Neuroscience Research has been a driving force behind this collaborative endeavor [12, 13]. It serves as a framework for coordinating neuroscience research efforts and aims to promote collaboration across centers in data sharing and tackling challenges of the brain and its disorders. Also, a group of European institutes and funders supported the establishment of large-scale biomedical projects and databases to collect genetic and other types of data from a diverse range of populations since 2006 [14]. This database provides researchers with a wealth of data to investigate the relationship between genetic factors, lifestyle choices, environmental exposures, and various health outcomes. In this section, we will highlight several outstanding large-scale neuroimaging projects and explore the factors contributing to their success.

Given the variety of project types, we focused on different population-based projects that fall into several categories. These include projects centered around healthy populations, those targeting specific diseases, and composite platforms that integrate various data sources. In our description, we first introduced the projects and basic information about the databases, followed by a discussion of significant discoveries made possible by these projects in their respective fields. In Table 1, we offered a more detailed summary of the different types of projects, focusing primarily on those with cohort sizes of around 1000 or more. The table includes specific details such as demographic characteristics, data types, imaging modalities, and the update status of each project.

**Table 1:** Summary of key neuroimaging projects

Project type	Project	Cohort Size	Participants	Gender	Age distribution	Ethnicity	Data types	Imaging modalities	Data accessibility	Funding Agency	Update Status	Data Collection	Website
Health-based projects	HCP (human connectome project)	HCP young adult: 1200 HCP lifespan: 4500 HCP disease-related: 4000 + (varias accroding to projects)	Healthy subjects	Varies	Varies	Predominantly White	Imaging data Genetic data Clinical assessment	Structural MRI diffusion MRI Functional MRI MEG ASL	Open access with registration	1.NIH (NIMH, NINDS) 2. McDonnell Foundation 3. Others	Completed (main phase)	Longitudinal	https://www.humanconnectome.org/
	UK Biobank	60,000+	General population	Roughly equal male/female	40–69	Broadly representative of the UK population	Imaging data Genetic data Clinical assessment Health linkages Biomarkers	Structural MRI diffusion MRI Functional MRI MEG EEG ASL	Request submitting	1. Wellcome trust 2. Medical research council 3. Department of health 4. Scottish government 5. The Northwest regional development agency 6. Others	Ongoing, data updated regularly	Longitudinal	https://www.ukbiobank.ac.uk/
	Healthy brain network	~5000	Children/adolescents healthy adults	Roughly equal male/female	5–21	Diverse, Primarily U.S.	Imaging data Clinical assessment Genetic data Envirionmenal data	Structural MRI diffusion MRI Functional MRI EEG	Request submitting	1. Child mind institute 2. Individual donors and private foundations	Ongoing, data updated regularly	Cross-sectional	https://healthybrainnetwork.org/
	OASIS (open access series of imaging studies)	OASIS-1: 416 OASIS-2: 150 OASIS-3: 1379 OASIS-TAU: 451 OASIS-4: 663	Healthy adults AD patients	Roughly equal male/female	20–90+	Predominantly white	Imaging data Clinical assessment Biomarkers	Structural MRI Diffusion MRI Functional MRI PET -CT	Request submitting	1. NIA 2. Alzheimer’s Association	Ongoing, data updated regularly	Cross-sectional (OASIS-1 and OASIS-4) Longitudinal (OASIS-2 and OASIS-3)	https://www.oasis-brains.org/
	(GSP) Brain genomics superstruct project	1570	Healthy adults	Roughly equal male/female	10–35	Diverse, primarily U.S.	Imaging data Clinical assessment Genetic data	Structural MRI diffusion MRI Functional MRI	Request submitting	1. NIH 2. Harvard Universirty 3. Massachusetts General Hospital 4. Others	Completed	Cross-sectional	https://www.neuroinfo.org/gsp/
	ABCD (adolescent brain cognitive development)	~11,880	Children/adolescents	Roughly equal male/female	9–10 at baseline	Diverse, primarily U.S.	Imaging data Clinical assessment Genetic data Envirionmenal data	Structural MRI diffusion MRI Functional MRI	Open access with registration	1. NIH (NIMH, NINDS, and NICHD)	Ongoing, data updated regularly	Longitudinal	https://abcdstudy.org/
	CHCP (Chinese human connectome project)	366	Healthy adults	Roughly equal male/female	18–80	Chinese Han	Imaging data Clinical assessment	Structural MRI diffusion MRI Functional MRI	Open access with registration	1. Beijing Municipal Science and Technology Commission 2. Chinese Institute for Brain Research (Beijing) 3. National Natural Science Foundation of China 4. Ministry of Science and Technology of China	Ongoing, data updated regularly	Cross-sectional (up to now)	https://www.scidb.cn/en/detail?dataSetId=f512d085f3d3452a9b14689e9997ca94
Disease based projects	ADNI (Alzheimer’s disease neuroimaging initiative)	ADNI1:800 ADNI GO: 200 new ADNI2: 650 new ADNI3: 500–1200 new ADNI4: 500 new	Healthy adults, Mild cognitive impairment patients AD patients	Roughly equal male/female	55–90	White Hispanic/Latino	Imaging data Clinical assessment Genetic data Biospecimen	Structural MRI diffusion MRI Functional MRI PET	Open access with registration	1. NIA 2. NIBIB 3. Alzheimer’s Association 4. Others	Ongoing, ADNI-4 now	Longitudinal	https://adni.loni.usc.edu/
	TCIA (the cancer imaging archive)	20+ brain tumor-related projects (sample size varies according to projects)	Tumors (glioma, meningoma, medulloblastomas, etc.)	Varies	Varies	Varies	Imaging data Clinical assessment Genetic data Pathology	CT structural MRI diffusion MRI PET	Open access with registration (some need to submit requests)	1. National Cancer Institute 2. Some non-governmental organizations 3. Public–private partnerships	Ongoing, data updated regularly	Some are cross-sectional	https://www.cancerimagingarchive.net/
	BraTS (the brain tumor segmentation)	~2000	Tumors	N/A	N/A	N/A	Imaging data Genetic data	Structural MRI	Free Downloading	Medical image computing and computer-assisted intervention	Ongoing, data updated regularly (2023 now)	Cross-sectional	http://braintumorsegmentation.org/
	PPMI (Parkinson’s progression markers initiative)	~1800	Healthy adults PD patients Prodormal PD	Roughly equal male/female	30–90	Predominantly White	Imaging data Clinical assessment Genetic data	Structural MRI diffusion MRI Functional MRI SEPCT	Request submitting	1. Michael J. Fox Foundation for Parkinson’s research 2. Aligning science across Parkinson’s	Ongoing, data updated regularly	Longitudinal	https://www.ppmi-info.org/
	ABIDE (the Autism brain imaging data exchange)	2156 (ABIDE-I:1112, ABIDE-II: 1044)	Healthy controls Austim patients	Roughly equal male/female	5–64	Predominantly White	Imaging data Clincial assessment	Structural MRI diffusion MRI Functional MRI	Open access with registration	1. NIMH 2. Child Mind Institute 3. Others	Completed	Cross-sectional	https://fcon_1000.projects.nitrc.org/indi/abide/
	ADHD200 (attention deficit hyperactivity disorder-200)	~950	Helathy children/adolescents ADHD patients	Roughly equal male/female	7–21	Diverse, primarily U.S.	Imaging data Clinical assessment Genetic data	Structural MRI Functional MRI	Open access with registration	NIH	Completed	Cross-sectional	https://fcon_1000.projects.nitrc.org/indi/adhd200/
Data resources	ENIGMA	Varies according to different projects											https://enigma.ini.usc.edu/
	Human brain projects												https://www.humanbrainproject.eu/
	OpenNeuro												https://openneuro.org/
	OpenfMRI												http://openfmri.org/dataset/
	NeuroSynth												https://neurosynth.org/

*NB: ENIGMA* enhancing neuroimaging genetics through meta-analysis, *N/A* not applicable, *NIA* the National Institute of Aging, *NIBIB* the National Institute of Biomedical Imaging and Bioengineering, *NICHD* the Eunice Kennedy Shriver National Institute on Child Health and Human Development, *NIH* National Institute of Health, *NIMH* the National Institute of Mental Health, *NINDS* the National Institute of Neurological Disorders and Stroke

### Human connectome project (HCP)

In 2010, the NIH allocated approximately $40 million [15] in funding to two consortia as part of the HCP [16] (https://www.humanconnectome.org/). The objective was to expedite advancements in neuroimaging methods and produce high-quality data to enhance our understanding of the connections between different regions of the healthy adult brain. A collaboration between Washington University, the University of Minnesota, and Oxford University formed the “WU–Minn–Ox” consortium for mapping structural and functional connectivity and to investigate relationships between behaviors and lifestyle [17, 18]. Another “MGH-UCLA” consortium, centered at Massachusetts General Hospital and the University of California at Los Angeles, focused on creating a 3-T MRI scanner with a high magnetic field gradient (300 mT/m) strength used for diffusion imaging [19, 20].

The initial target of HCP was to collect around 1200 healthy adults (ages 22–35). The project aimed at establishing standardized protocols and ensuring high-quality data collection of multiple modal images, including structural MRI, task-functional MRI (task-fMRI), resting-state fMRI (rs-fMRI), diffusion MRI (dMRI), and MEG for each subject. Apart from data acquisition, the standard data preprocessing protocols were also designed and released as a reference workflow named HCP Pipelines for multimodal data analysis to ensure reproducibility and reliability.

In 2016, the young adults S1200 dataset with a new multi-modal brain parcellation [21] was released, with 184 subjects scanned by a 7-T MRI scanner and 95 subjects tested by resting-state MEG. The HCP parcellation delineated 180 areas per hemisphere, and this parcellation scheme has since gained widespread recognition and acceptance as an important tool for brain network connection analysis [22].

To extend the HCP, the NIH also promoted Lifespan projects [23, 24] since 2016. These projects extend the range of sample size and ages of healthy populations. They provide more reliable resources for researchers to explore cross-sectional differences and longitudinal changes across different stages of the lifespan.

### UK Biobank

UK Biobank (https://www.ukbiobank.ac.uk/), one of the largest population-based prospective projects, was established to collect and analyze genetic and nongenetic information from a diverse population in the United Kingdom [25, 26]. It aims to gather valuable data that can contribute to a better understanding of various health conditions and their underlying genetic and environmental factors, followed by improving human health.

As part of this initiative, UK Biobank has successfully collected imaging data from over 60,000 subjects to date, with a target of 100,000. More than 3000 subjects among them have gone through a test–retest MRI scan at 2-year intervals. These imaging data encompass various modalities and provide a wealth of information for researchers to investigate the links between genetics, environment, lifestyle, and disease. Also, the database of the UK Biobank offers imaging-derived phenotypes (IDPs) such as thickness or volume of gyrus, functional connectivity, and other metrics, which could assist researchers in linking the association between these IDPs to genetic status or lifestyle factors [27].

As an illustration of the prospective nature of UK Biobank, researchers have leveraged the test–retest imaging data from patients who have experienced COVID-19. By comparing IDPs before and after virus infection, they were able to identify longitudinal effects associated with the virus including reductions in gray matter thickness, and tissue damage in regions connected to the primary olfactory cortex [28]. The UK Biobank also provides other clinical, regional, and genetic information to associate the risk factors with various diseases [29]. These findings highlight the value of large-scale prospective projects in studying the long-term effects of diseases and identifying potential biomarkers or indicators of disease progression.

Since the establishment of the UK Biobank, more than 10,000 researchers have registered to access and utilize the data available; beyond that, over 700 institutes worldwide have published papers based on analyses and findings derived from the data provided by the UK Biobank. This wide-reaching publication activity showcases the broad collaboration and engagement of researchers from diverse institutions across the globe [30].

### Chinese human connectome project (CHCP)

While the HCP and UK Biobank have provided high-quality multi-modal images, the participants in these projects have predominantly come from Western societies, primarily Europe and America. Consequently, the generalizability of research findings based on these datasets to Asian populations may be limited. Cultural and genetic differences, as well as variations in environmental factors, can significantly impact brain function and connectivity [31].

In 2017, the CHCP (https://www.Chinese-HCP.cn) was established by Gao and his team [5]. The current release represents almost 400 healthy Han Chinese adults living in Eastern culture. The release utilizes consistent collection and processing protocols for the HCP, ensuring compatibility and comparability between the datasets. Although the current number of participants in CHCP is still limited, we believe that this project, which focuses on the Han Chinese population, is highly valuable for the broader research community in China.

However, the CHCP is a relatively new initiative with some limitations that need to be addressed and improved upon. (1) The current sample size still needs to be expanded to increase the statistical power and representativeness of the findings; (2) the focus is mainly on participants who may not fully represent the entire Chinese population. Collaborating with research institutions from various regions to ensure a broader representation of different cultural and ethnic backgrounds is necessary; and (3) longitudinal and prospective research that collects test–retest scans over time is essential to investigate the dynamics of the brain [32].

### Adolescent brain cognitive development (ABCD)

The ABCD (https://abcdstudy.org/) mainly focuses on brain development and child health in the U.S. [33]. It has collected multiple modal images from more than 12,000 children, ages 9–10, using various scanners, as well as physical, cognitive, social, emotional, environmental, behavioral, and academic assessments. This project has opened new avenues for research in cognitive neuroscience, particularly in relation to young children and adolescents.

The 5^th^ ABCD Data Release published in 2013 also contained more follow-up data, such as test–retest MRI scans and school performance data. By giving the unique opportunity to assess the physical and mental development of children, the ABCD expanded the research field of cognitive neuroscience, facilitating the attribution to young children and adolescents. For example, Scott et al [34] utilized the resting-state functional connectivity (RSFC) of 2188 participants of the ABCD dataset and found reproducible functional network architecture across individuals. They identified correlations between reproducible RSFCs and behaviors, as well as cognition. Furthermore, researchers could utilize the abundant data to investigate the relationships between brain IDPs and various developmental disorders, including mental or psychiatric illness [35, 36], drug and alcohol addiction [37], and other related conditions.

### Alzheimer’s disease neuroimaging initiative (ADNI)

Founded in 2004 by the National Institute on Aging (NIA) and the National Institute of Biomedical Imaging and Bioengineering (NIBIB), ADNI (https://adni.loni.usc.edu/) is a world-influenced project aiming at understanding and tracking the progression of Alzheimer’s disease (AD) through neuroimaging, clinical assessment and other biomarker data [38–40]. Currently, ADNI has gone through four phases of development, and has collected longitudinal multi-omics data from thousands of AD patients and healthy controls. Using its data, studies have found that RSFCs in AD are associated with the accumulation of tau pathology [41]. Also, by combining rs-fMRI and tau- Positron Emission Tomography (PET), researchers can potentially provide robust evidence for cognitive impairment and prognosis prediction in AD patients [41–43].

Furthermore, cortical and subcortical RSFCs could serve as strong biomarkers to reveal cognitive and behavioral performance, which could promote precision diagnosis and early intervention [44–46]. ADNI projects have continuously provided substantial data support, enabling researchers from different regions to share data and conduct large-scale population surveys and follow-ups. Through collaborative efforts of researchers, the data quantity of ADNI continues to expand, and the research content continues to deepen, providing effective evidence for the screening, diagnosis, and treatment of AD.

### The cancer imaging archive (TCIA)

TCIA (https://www.cancerimagingarchive.net/) was founded in 2011 under the fund of the Cancer Imaging Program, a part of the National Cancer Institute (NCI), and hosts a large release of medical imaging data on cancers. It already has more than 30 million images of different modalities, and some projects even have pathological images. Also, because both TCIA and The Cancer Genome Atlas (TCGA) were supported by NCI, participants who have radiomics data, can also have their corresponding genomic and pathological data collected. This allows for multi-omics analysis, where different data types are integrated and analyzed together [47–50].

TCIA neuroimaging projects provide over 20 datasets, including collections of gliomas, meningiomas, medulloblastomas, and others. Specifically for gliomas, TCIA includes structural imaging corresponding to different molecular subtypes, allowing users to extract radiomics or radiogenomics features for analysis [48, 51]. Among them, UPENN-GBM [52] and UCSF-PDGM [53] are the two largest glioma projects, each of which provides over five hundred subjects with structural and diffusion MRI data for connectome study. Using these resources, sophisticated AI models for tumor classification, segmentation, and genomics prediction would be created to assist in clinical practice and medical education [54].

### Enhancing neuroimaging genetics through meta-analysis (ENIGMA)

Beginning in 2009, the ENIGMA (https://enigma.ini.usc.edu/) is now the oldest and largest neuroimaging genetics consortia, with more than 50 working groups across the world [55, 56], including major depressive disorder [57, 58], schizophrenia [59], obsessive-compulsive disorder [60, 61], stroke [62], and other neuropsychiatric diseases via analysis of genome-wide association studies, and neuroimaging metrics [63]. The ultimate goal is to establish neuro-genetic connection networks that can shed light on the intricate relationships between genetics, brain function, and various brain disorders.

Unlike the projects that collect brain imaging data as part of their comprehensive datasets, ENIGMA operates as a collaborative platform that brings together researchers worldwide to conduct analyses on these data. It aims to pool data from different studies and research groups to increase sample sizes, enhance statistical power, and uncover robust associations between brain imaging measures across various populations and disorders [64]. Due to the collaborative research patterns, there are several special statistical designs for ENIGMA. One approach involves a meta-analysis, pooling subjects and results across studies as a standard protocol and data harmonization [65, 66]. By collecting all individual-participant data into one statistical model, the results will be more generalized, leading to a more comprehensive conclusion due to its consideration of data miss or bias, study-specific differences, and other potential sophisticated conditions [60, 65, 67], however, at the cost of designing more complex statistical models and incurring higher computational expenses. Besides combining the neuroimaging and genetic data into a large dataset for analysis, it also allows for volume- or surface-based analysis.

### Others

For the sake of brevity, we only listed a small fraction of the large-scale neuroimaging projects available. Some projects focus on systematic data collection from population samples, with regular tracking and follow-up, aiming at building integrated data covering the lifespan of individuals, including the Human Brain Projects (HBP) [68], Open Access Series of Imaging Studies (OASIS) [69], Cambridge Centre for Ageing and Neuroscience (Cam-CAN) [70, 71], Brain Genomics Superstruct Project (GSP) [72] and the Chinese Imaging Genetics (CHIMGEN) [6], etc. Others, such as Parkinson’s Progression Markers Initiative (PPMI) [73], Autism Brain Imaging Data Exchange (ABIDE) initiative [74], and Attention Deficit Hyperactivity Disorder-200 (ADHD-200) projects [75], mainly concentrate on specific diseases or some physical or mental conditions, providing data support for their diagnosis, treatment, and research. Moreover, there are still several open platforms like OpenNeuro (https://openneuro.org/), OpenfMRI (http://openfmri.org/dataset/), and NeuroSynth (https://neurosynth.org/), which provide different modalities of neuroimages as Brain Imaging Data Structure (BIDS) or synthesis for researchers to focus on mechanisms of cognition and behavior, and other projects.

Although these research projects and datasets have their own focuses, we must acknowledge that there are significant differences in terms of funding support, research capacity, data collection methods, and data types between different countries or research institutions. Furthermore, we also found that many of these projects were established by organizations in developed regions, thus, for some developing countries, further enhancement of project management and implementation capabilities is still needed.

## Experience from these projects

### Project construction and management principles

It is crucial to establish robust infrastructure at all stages for large-scale projects, including data collection protocols, standardized imaging processing techniques, ethical guidelines, etc. [76]. Firstly, during the initial design phases of project planning, establishing well-defined inclusion and exclusion criteria and determining the specific types of data to be collected is essential. Drawing from the experience of these projects, researchers should carefully determine the specific data modalities to be collected. Striking a balance between project requirements and available resources is often the cornerstone of successful project management [77].

To achieve this balance, project managers must conduct a thorough analysis of the project’s requirements and assess the available resources. This is particularly important for studies with a clinical focus, as overlooking or neglecting certain indicators or features during planning may necessitate spending significant time and effort going back and collecting them. For example, Stroganov et al [78] proposed a curation and pipeline for UK Biobank data integration and harmonization, improving data qualities.

Secondly, it is important to implement standardized training for project participants. This training should cover effective communication techniques with participants, ensuring the protection of their privacy, and establishing standardized protocols for the collection of data using scales, questionnaires, or other instruments, together with long-term follow-up. Following these standardized procedures will ensure consistency and accuracy in data collection across different sites, thereby enhancing the reliability and validity of the collected data.

Thirdly, standardized data structure data acquisition pipelines, and quality control measures are essential for project management [76], particularly in projects involving multiple centers, modalities, and sequences [79]. Usually, BIDS [80] is utilized as a standardized format for storing, organizing, and sharing data with multiple modalities [81]. As for data acquisition, in projects including the HCP, UK Biobank, ABCD, ADNI, etc., there are clear protocols available on the official websites for reference. These protocols contain detailed information on scanning machines, parameters, modalities, and even non-imaging data collection methods, and are regularly updated. Moreover, to protect the privacy of subjects, anonymization, and de-identification procedures should be considered as a regulation during the processing stages of the images and clinical information. These projects implemented detailed anonymization processes. Personal information has been removed, and the associated clinical information has also been anonymized when accessing their public data. At present, different deep learning frameworks and approaches have been implemented for anonymization [82, 83].

However, we have noticed that the major large-scale projects in neuroscience primarily focus on brain development, aging, and some neurodegenerative or psychiatric disorders. There seems to be less emphasis on neurosurgical conditions, particularly brain tumors. Although there are some datasets related to brain tumors [84, 85], there is still a scarcity of large-scale multi-modal imaging and clinical data. Brain tumors, especially those located in functional areas, can lead to varying degrees of brain tissue invasion, compression, and subsequent abnormalities in functional activity. TCIA, or BraTS [86, 87] contains lots of tumor data, but the tumor-related connectome exploration value is limited due to imaging modality and the lack of assessments of various clinical indicators such as language, emotion, and behavior. Therefore, for neurosurgeons, efforts are now being directed towards establishing neuro-oncological multi-modal imaging projects to provide robust population evidence for brain tumor-related neuro-cognitive research.

In summary, in the sense of project establishment, conducting large-scale projects faces many obstacles, but it is becoming increasingly necessary and beneficial. The motivation extends beyond securing funding support or publishing papers; the deeper aims are to promote collaboration across regions, especially developing countries, and improve medical care for patients who suffer from pain and diseases.

### The rules and guidelines for data requests and usage

As users of these datasets, there are rules and regulations that need to be learned and understood. These rules are in place to protect the privacy and confidentiality of the participants and to ensure ethical and responsible use of the information.

First of all, when applying for data, users should provide accurate information and carefully read the informed consent documents. Data from sources from HCP, OpenNeuro, BraTS, TCIA, etc. can be accessed directly, however, projects like ADNI, UK-biobank, ENIGMA, and other disease-related datasets often require applicants to submit a research proposal to assess the suitability and alignment of the proposed study. They should state the amount of data, and its intended use, and even provide a clear introduction to the project. These steps are crucial because users may come from different parts of the world, and project managers need to assess whether project information and participant privacy will be safeguarded [88]. Users should also comply with any restrictions or guidelines and maintain the confidentiality of the data, refrain from disclosing or sharing the data with other entities or researchers without proper authorization.

Furthermore, another important consideration is to select appropriate processing pipelines. Pipelines are essential for preprocessing and analyzing the data in a consistent and standardized manner. The main focus is aligning them with specific research questions. Some may offer predefined pipelines, but more commonly, some may only have the imaging data with BIDS or even the raw format. Researchers could adapt their analysis steps based on these workflows to meet their research needs.

A bunch of data analysis tools have been released for preprocessing, including traditional tools like FSL [89], SPM [90], and AFNI [91], etc. [92, 93], integrated tools such as fMRIprep [94], C-PAC [95, 96], and others [97, 98]. It is still an argument about which software or pipelines are better [99, 100]. Indeed, we think the choice of processing pipelines should consider factors such as the research platform’s capabilities, the number of participants, the need for multitask processing, and so on.

In addition, sample size impact must be considered an important factor when utilizing the datasets [101]. The appropriate sample size depends on factors including the effect size of interest, data variability, desired statistical power, and analysis complexity. And it should be clearly declared during the projects [102]. In fact, the estimation of sample size might be practically difficult in some rare disease studies. A recent study [103] revealed that population-based brain-wide association studies require as many participants as possible to get more stable and replicable results. Smaller sample sizes, however, are also important for providing blueprints for reducing MRI artifacts, as well as improving the amount of data.

**Fig. 1:**
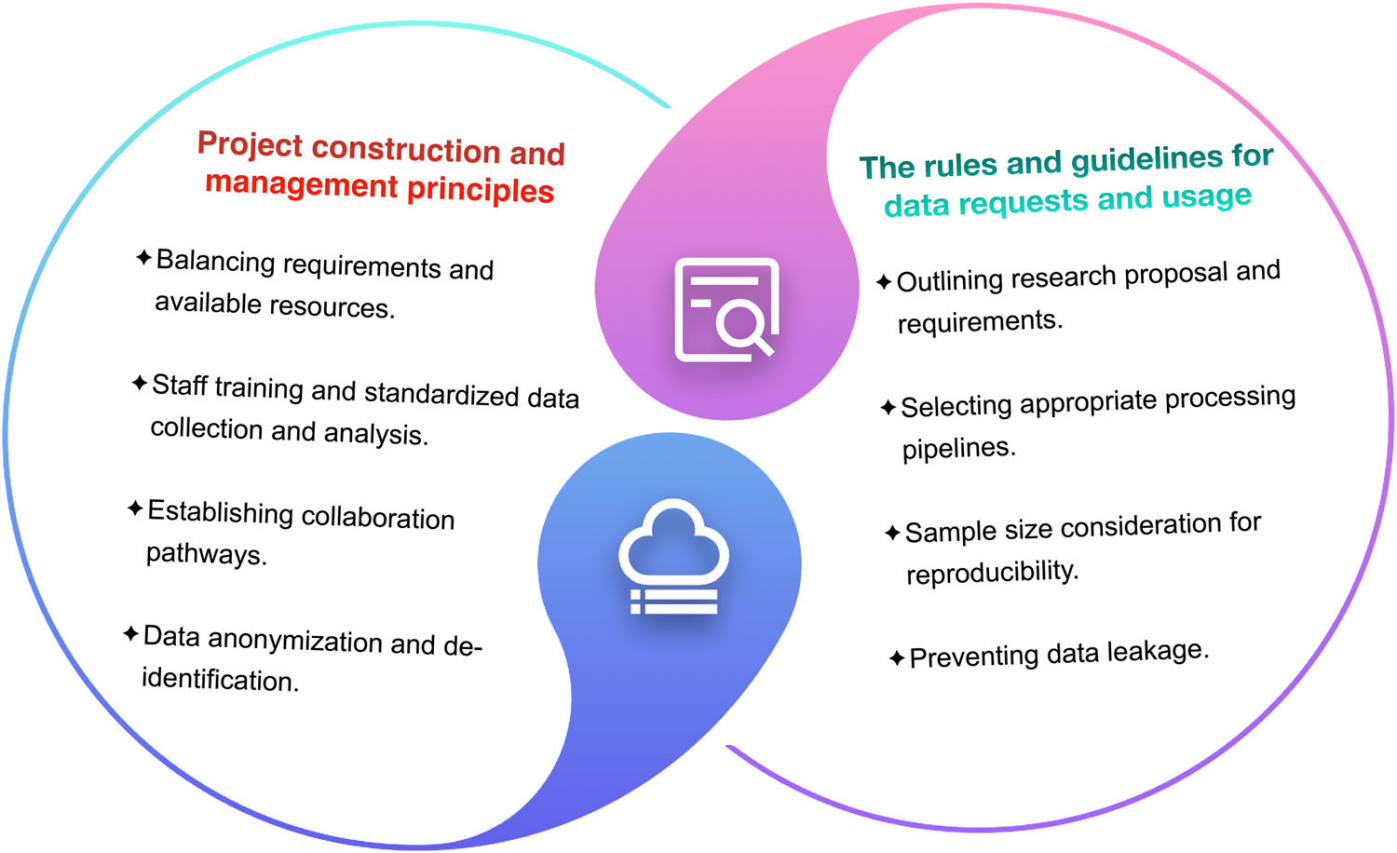
An overview of the experience from these neuroimaging projects, including the principles for project construction and guidelines for data requests

In the above discussion, we investigated the considerations and guidelines for both the project builders and users. These are often established conventions based on the collective experience and knowledge of the involved parties. In the next section, we will focus on the ethical and legal aspects and discuss the challenges and issues associated with data releasing and sharing, and privacy protection (Fig. 1).

## Ethical and privacy issues

Data sharing and releasing is a growing trend with the increasing volume of generated data. However, this trend also brings along a range of ethical and legal concerns and issues that need to be addressed. Data governance is defined as the principles, frameworks, and policies to maintain safety and privacy during all stages of data life [7].

### Ownership

Before analyzing and sharing data, it is essential to establish data ownership. This is especially important in clinical projects involving various departments, including clinical departments like neurology, neurosurgery, and psychiatry, allied departments like radiology, and some third parties like funding agencies and collaborative institutions. Should there be conflicts about data ownership, the first responsible units of the subjects should have primary control over their raw data, regardless of previous consent. The consent should include clear information about data usage, anonymization, and privacy protection concerns, and provide options for data withdrawal or opting out of certain analyses, ensuring transparency in data handling practices.

The general data protection regulation (GDPR) (https://gdpr-info.eu/), drafted and passed by the European Union (EU), encompassed the “Rights of data subjects”. It outlined specific provisions regarding the protection and management of personal data, ensuring participants’ privacy and control over their own information, as well as obligating the researchers to respect and protect the participants and their data [104]. As for the subsequent results derived from the original data, they are primarily the responsibility of the main authors or creators [105].

Additionally, in cases where multiple individuals or departments have contributed to the data analysis process, building agreements and guidelines before and during the projects is necessary. While decentralized data management is a growing trend [106], it is vital to consider the context in developing regions where researchers are affiliated with specific hospitals or institutions. It is recommended that these institutions adopt a coordinated approach to project management in situations where different departments or divisions have conflicting interests. This will help ensure a more cohesive and effective process for managing the project.

### Policies and regulations

To foster a better environment for data usage and sharing, policies, and legislation are essential in establishing a framework that governs data collection, storage, and sharing, ensuring the protection of individual privacy and rights. The Department of Health and Human Services, the National Institute of Mental Health (https://grants.nih.gov/grants/guide/notice-files/NOT-MH-23-100.html), and NIH (https://grants.nih.gov/grants/guide/notice-files/NOT-OD-21-013.html) have indeed established their policies and regulations to govern the acquisition and sharing of data in research projects [88, 107]. These policies aim to ensure compliance with ethical standards, privacy protection, and responsible data management practices. Researchers are required to adhere to these rules throughout the entire data lifecycle.

Some countries and regions have specific data protection laws in place, such as the GDPR in the EU and the California Consumer Privacy Act (https://oag.ca.gov/privacy/ccpa) in the U.S. [108], the Health Insurance Portability and Accountability Act (https://www.hhs.gov/hipaa) in the U.S. [109], and the Personal Information Protection and Electronic Documents Act (https://laws-lois.justice.gc.ca) in Canada [110], etc. These regulations and laws define the rights of individuals regarding their data and set obligations for organizations handling such data.

Institutions in Asian countries like the Ministry of Education, Culture, Sports, Science and Technology (https://www.mext.go.jp/en/) and the Japan Agency for Medical Research and Development (https://www.amed.go.jp/en/) in Japan, the National Health Commission (http://www.nhc.gov.cn/) and the Chinese Academy of Sciences (https://www.cas.cn/) in China, etc. have also developed related policies and regulations for data management. Recently, Shen et al [111], from the perspective of law, also illustrated the challenges and detailed recommendations for policy and ethical challenges for neuroimaging research.

However, current policies mainly address data management within institutions, with limited emphasis on broad collaboration across institutions and borders. Encouraging cross-institutional partnerships and establishing mechanisms for international data harmonization can enhance scientific research, foster innovation, and address global health challenges more effectively. Developing countries should recognize the potential benefits of collaboration and work to create policies that enable responsible and secure data-sharing on a broader scale. Ultimately, a multi-layered approach combining technical measures, policy frameworks, and collaborative efforts is necessary to strengthen data regulation and protect against theft and leaks across sectors and institutions.

### Ethics related to AI

At present, state-of-the-art AI applications have made significant advancements in assisting clinical practice and research exploration. They have been utilized in various areas such as lesion detection, prognosis prediction, and drug development [112, 113]. These AI-driven techniques offer valuable support to healthcare professionals, enhancing patient care and medical research.

However, some concerns and ethical issues need to be addressed. Firstly, AI models, especially some deep learning models, often operate as ‘black boxes’, making it challenging to understand how they arrive at decisions. Efforts should be made to develop transparent and interpretable AI models so that they can provide clear explanations for their predictions and recommendations [114].

Secondly, determining accountability and responsibility in cases where AI models make erroneous or harmful decisions is a significant ethical concern [115]. AI-driven decisions should be maintained and supervised by human physicians, and clearer guidelines should be established to allocate responsibility and address any potential negative consequences.

Thirdly, concerns arise regarding ownership, validation, reliability, publication ethics, etc., when dealing with data created by generative AI models [116]. Furthermore, AI can inadvertently perpetuate biases present in the data used for training [117, 118]. Thus, collaboration across the globe is essential to train more generalized models for clinical application.

In summary, the idea of human uniqueness and centrality has shaped many aspects of our ethical, legal, and social systems. With the improvement of AI and its capabilities, it is becoming imperative to adapt our policies and safeguards to address the evolving landscape.

### Project lifecycle management

Project lifecycle management refers to the comprehensive oversight and control of a project from initiation to closure. By following a structured and systematic approach to project lifecycle management, project managers and related institutions can improve project success rates, enhance collaboration, and optimize resource utilization.

In clinical cases, Hospital Information Systems combined with Picture and Archival Communication Systems provide a foundational and centralized database for clinical management [119]. However, for project management, there are some different components that aim at achieving project objectives efficiently and effectively, and these include ethics and some other issues that need to be addressed.

During the lifecycle of a project, the management team has to build standard operating procedures (SOPs), which describe the full standardized stages of the project from planning and data collection to data sharing and research publishing [81, 120]. We could draw and learn from the successful projects mentioned and form a project management team with experts in neuroscience, data science, computer science, ethics, etc. This team can collaborate to develop comprehensive and clear SOPs, with regular discussions and updates to promote the progress of the project.

Before initiation, pre-registration is crucial. It involves submitting the study design, hypotheses, sample size, and analysis plan to a recognized platform or registry before data collection and analysis begin [121], especially for clinical trials. More publishing bodies require the pre-registration report for peer review to distinguish confirmatory research (testing pre-specified hypotheses) from exploratory research (generating new hypotheses), and to promote transparency and accountability through public supervision [122, 123]. The platforms most commonly used for pre-registration include OSF (https://osf.io/), aspredicted.org, clinicaltrals.gov and chictr.org.cn, etc.

During the processing stages, regular project monitoring and ethical review are essential to track milestones, identify potential issues, and make necessary adjustments. To facilitate better project quality control, implementing measures to prevent errors, defects, and deviations from the project’s objectives and ensuring that deliverables and outcomes meet the expected levels of quality should be considered.

An international data governance framework should be established to address data-related challenges, ensuring data privacy and security, promoting responsible data use, and fostering effective international collaboration and data-driven initiatives [7, 10].

## Discussion and perspective

Based on our previous discussion, it is evident that establishing large-scale population-based projects and building comprehensive neuroimaging databases are the current trends and a crucial pathway for the future development of neuroscience and interdisciplinary applications, both at the institutional and individual researcher levels.

At the institutional level, organizations are increasingly recognizing the potential of large-scale projects involving diverse populations. These projects enable the collection of extensive and diverse data, enhancing the robustness of research outcomes. Building partnerships and collaborations across disciplines would provide a communication bridge and network for outstanding research projects. However, these will not be without several challenges. For example, inter-organizational conflicts of interest that should be balanced can arise, especially in regional collaborations or within departments [124, 125].

While protecting the rights of research participants, it’s equally important to ensure fair benefits for those who contribute significantly to the projects. Developing countries and regions can learn from these advanced project management teams and promote the progress of their own projects through policy, legal, and managerial efforts. To regulate third-party interests, establishing effective governance mechanisms and ensuring alignment with ethical standards are necessary and of utmost importance.

Simultaneously, researchers themselves play a critical role in driving these initiatives forward. They must actively participate in project design, data collection, and analysis. On one hand, researchers need to move beyond focusing solely on individual diseases, embrace multidisciplinary collaborations, utilize large-scale datasets, and explore individualized analysis. On the other hand, this also places greater expectations on researchers. To achieve a more profound understanding of diseases, researchers may need to enhance their expertise in various fields, including neuroscience, bioengineering, and even AI fields. Also, they have to collect and organize cases that meet the inclusion criteria and regularly follow up on these cases. The focus should be on integrating research topics with actual work rather than collecting data just for the sake of it.

## Conclusion

In this review, learning from advanced neuroimaging and neuroscience projects, we discussed the importance and necessity of establishing large-scale projects and comprehensive databases and pointed out issues that are essential to establishing and utilizing such projects. This is a promising direction that requires collaboration across multiple disciplines, adherence to standardized procedures, state-of-the-art technological assistance, and ethical management. These efforts will not only advance our understanding of the human brain but also improve innovation and patient care in clinical settings from a wider perspective.

## Abbreviations

ABCD: Adolescent brain cognitive development
ADNI: Alzheimer’s disease neuroimaging initiative
AI: Artificial intelligence
BIDS: Brain imaging data structure
CHCP: China human connectome project
ENIGMA: Enhancing neuroimaging genetics through meta-analysis
HCP: Human connectome project
IDP: Imaging-derived phenotype
MRI: Magnetic resonance imaging
NIH: National Institutes of Health
SOP: Standard operating procedures

## References

[1] de Schotten MT, Croxson PL, Mars RB (2019) Large-scale comparative neuroimaging: Where are we and what do we need? Cortex 118:188–202. 10.1016/j.cortex.2018.11.028 30661736 10.1016/j.cortex.2018.11.028 PMC6699599

[2] Surianarayanan C, Lawrence JJ, Chelliah PR, Prakash E, Hewage C (2023) Convergence of artificial intelligence and neuroscience towards the diagnosis of neurological disorders—a scoping review. Sensors (Basel). 10.3390/s23063062 10.3390/s23063062 PMC10053494 36991773

[3] Basso MA, Frey S, Guerriero KA et al (2021) Using non-invasive neuroimaging to enhance the care, well-being and experimental outcomes of laboratory non-human primates (monkeys). Neuroimage 228:117667. 10.1016/j.neuroimage.2020.117667 33359353 10.1016/j.neuroimage.2020.117667 PMC8005297

[4] Messinger A, Sirmpilatze N, Heuer K et al (2021) A collaborative resource platform for non-human primate neuroimaging. Neuroimage 226:117519. 10.1016/j.neuroimage.2020.117519 33227425 10.1016/j.neuroimage.2020.117519 PMC9272762

[5] Ge J, Yang G, Han M et al (2023) Increasing diversity in connectomics with the Chinese human connectome project. Nat Neurosci 26:163–172. 10.1038/s41593-022-01215-1 36536245 10.1038/s41593-022-01215-1

[6] Xu Q, Guo L, Cheng J et al (2020) CHIMGEN: a Chinese imaging genetics cohort to enhance cross-ethnic and cross-geographic brain research. Mol Psychiatry 25:517–529. 10.1038/s41380-019-0627-6 31827248 10.1038/s41380-019-0627-6 PMC7042768

[7] Eke DO, Bernard A, Bjaalie JG et al (2022) International data governance for neuroscience. Neuron 110:600–612. 10.1016/j.neuron.2021.11.017 34914921 10.1016/j.neuron.2021.11.017 PMC8857067

[8] White T, Blok E, Calhoun VD (2022) Data sharing and privacy issues in neuroimaging research: opportunities, obstacles, challenges, and monsters under the bed. Hum Brain Mapp 43:278–291. 10.1002/hbm.25120 32621651 10.1002/hbm.25120 PMC8675413

[9] Bernier A, Molnar-Gabor F, Knoppers BM (2022) The international data governance landscape. J Law Biosci 9:lsac005. 10.1093/jlb/lsac005 35382430 10.1093/jlb/lsac005 PMC8977111

[10] Abraham R, Schneider J, vom Brocke J (2019) Data governance: a conceptual framework, structured review, and research agenda. Int J Inform Manage 49:424–438. 10.1016/j.ijinfomgt.2019.07.008

[11] Wilkinson MD, Dumontier M, Aalbersberg IJ et al (2016) The FAIR guiding principles for scientific data management and stewardship. Sci Data 3:160018. 10.1038/sdata.2016.18 26978244 10.1038/sdata.2016.18 PMC4792175

[12] Baughman RW, Farkas R, Guzman M, Huerta MF (2006) The National Institutes of Health Blueprint for neuroscience research. J Neurosci 26:10329–10331. 10.1523/JNEUROSCI.3979-06.2006 17035514 10.1523/JNEUROSCI.3979-06.2006 PMC6674678

[13] Mott MC, Austin CP, Bianchi DW et al (2019) The NIH blueprint for neuroscience research seeks community input on future neuroscience investments. J Neurosci 39:774–775. 10.1523/JNEUROSCI.2742-18.2018 30700523 10.1523/JNEUROSCI.2742-18.2018 PMC6382981

[14] Palmer LJ (2007) UK Biobank: bank on it. Lancet 369:1980–1982. 10.1016/S0140-6736(07)60924-6 17574079 10.1016/S0140-6736(07)60924-6

[15] News (2010) Human connectome project award $40 million. J Invest Med 58:929–935. 10.2310/JIM.0b013e3182025955

[16] Van Essen DC, Smith SM, Barch DM et al (2013) The WU–Minn human connectome project: an overview. Neuroimage 80:62–79. 10.1016/j.neuroimage.2013.05.041 23684880 10.1016/j.neuroimage.2013.05.041 PMC3724347

[17] Van Essen D (2014) The human connectome project: progress and prospects. Biol Psychiat 75:135s. s

[18] Elam JS, Glasser MF, Harms MP et al (2021) The human connectome project: a retrospective. Neuroimage 244:118543. 10.1016/j.neuroimage.2021.118543 34508893 10.1016/j.neuroimage.2021.118543 PMC9387634

[19] McNab JA, Edlow BL, Witzel T et al (2013) The human connectome project and beyond: initial applications of 300 mT/m gradients. Neuroimage 80:234–245. 10.1016/j.neuroimage.2013.05.074 23711537 10.1016/j.neuroimage.2013.05.074 PMC3812060

[20] Fan Q, Witzel T, Nummenmaa A et al (2016) MGH-USC human connectome project datasets with ultra-high *b*-value diffusion MRI. Neuroimage 124:1108–1114. 10.1016/j.neuroimage.2015.08.075 26364861 10.1016/j.neuroimage.2015.08.075 PMC4651764

[21] Glasser MF, Coalson TS, Robinson EC et al (2016) A multi-modal parcellation of human cerebral cortex. Nature 536:171–178. 10.1038/nature18933 27437579 10.1038/nature18933 PMC4990127

[22] Bryce NV, Flournoy JC, Guassi Moreira JF et al (2021) Brain parcellation selection: an overlooked decision point with meaningful effects on individual differences in resting-state functional connectivity. Neuroimage 243:118487. 10.1016/j.neuroimage.2021.118487 34419594 10.1016/j.neuroimage.2021.118487 PMC8629133

[23] Somerville LH, Bookheimer SY, Buckner RL et al (2018) The lifespan human connectome project in development: a large-scale study of brain connectivity development in 5–21-year-olds. Neuroimage 183:456–468. 10.1016/j.neuroimage.2018.08.050 30142446 10.1016/j.neuroimage.2018.08.050 PMC6416053

[24] Bookheimer SY, Salat DH, Terpstra M et al (2019) The lifespan human connectome project in aging: an overview. Neuroimage 185:335–348. 10.1016/j.neuroimage.2018.10.009 30332613 10.1016/j.neuroimage.2018.10.009 PMC6649668

[25] Sudlow C, Gallacher J, Allen N et al (2015) UK biobank: an open access resource for identifying the causes of a wide range of complex diseases of middle and old age. PLoS Med 12:e1001779. 10.1371/journal.pmed.1001779 25826379 10.1371/journal.pmed.1001779 PMC4380465

[26] Allen N, Sudlow C, Downey P et al (2012) UK Biobank: current status and what it means for epidemiology. Health Policy Techn 1:123–126. 10.1016/j.hlpt.2012.07.003

[27] Littlejohns TJ, Holliday J, Gibson LM et al (2020) The UK Biobank imaging enhancement of 100,000 participants: rationale, data collection, management and future directions. Nat Commun 11:2624. 10.1038/s41467-020-15948-9 32457287 10.1038/s41467-020-15948-9 PMC7250878

[28] Douaud G, Lee S, Alfaro-Almagro F et al (2022) SARS-CoV-2 is associated with changes in brain structure in UK Biobank. Nature 604:697. 10.1038/s41586-022-04569-5 35255491 10.1038/s41586-022-04569-5 PMC9046077

[29] Kolin DA, Kulm S, Christos PJ, Elemento O (2020) Clinical, regional, and genetic characteristics of Covid-19 patients from UK Biobank. PLoS One 15:e0241264. 10.1371/journal.pone.0241264 33201886 10.1371/journal.pone.0241264 PMC7671499

[30] Conroy M, Sellors J, Effingham M et al (2019) The advantages of UK Biobank’s open-access strategy for health research. J Intern Med 286:389–397. 10.1111/joim.12955 31283063 10.1111/joim.12955 PMC6790705

[31] Han S, Ma Y (2014) Cultural differences in human brain activity: a quantitative meta-analysis. Neuroimage 99:293–300. 10.1016/j.neuroimage.2014.05.062 24882220 10.1016/j.neuroimage.2014.05.062

[32] Noble S, Scheinost D, Constable RT (2021) A guide to the measurement and interpretation of fMRI test–retest reliability. Curr Opin Behav Sci 40:27–32. 10.1016/j.cobeha.2020.12.012 33585666 10.1016/j.cobeha.2020.12.012 PMC7875178

[33] Casey BJ, Cannonier T, Conley MI et al (2018) The adolescent brain cognitive development (ABCD) study: imaging acquisition across 21 sites. Dev Cogn Neurosci 32:43–54. 10.1016/j.dcn.2018.03.001 29567376 10.1016/j.dcn.2018.03.001 PMC5999559

[34] Marek S, Tervo-Clemmens B, Nielsen AN et al (2019) Identifying reproducible individual differences in childhood functional brain networks: an ABCD study. Dev Cogn Neurosci 40:100706. 10.1016/j.dcn.2019.100706 31614255 10.1016/j.dcn.2019.100706 PMC6927479

[35] Pornpattananangkul N, Leibenluft E, Pine DS, Stringaris A (2019) Association between childhood anhedonia and alterations in large-scale resting-state networks and task-evoked activation. JAMA Psychiatry 76:624–633. 10.1001/jamapsychiatry.2019.0020 30865236 10.1001/jamapsychiatry.2019.0020 PMC6552295

[36] Karcher NR, O’Brien KJ, Kandala S, Barch DM (2019) Resting-state functional connectivity and psychotic-like experiences in childhood: results from the adolescent brain cognitive development study. Biol Psychiatry 86:7–15. 10.1016/j.biopsych.2019.01.013 30850130 10.1016/j.biopsych.2019.01.013 PMC6588441

[37] Volkow ND, Koob GF, Croyle RT et al (2018) The conception of the ABCD study: from substance use to a broad NIH collaboration. Dev Cogn Neurosci 32:4–7. 10.1016/j.dcn.2017.10.002 29051027 10.1016/j.dcn.2017.10.002 PMC5893417

[38] Petersen RC, Aisen PS, Beckett LA et al (2010) Alzheimer’s disease neuroimaging initiative (ADNI): clinical characterization. Neurology 74:201–209. 10.1212/WNL.0b013e3181cb3e25 20042704 10.1212/WNL.0b013e3181cb3e25 PMC2809036

[39] Aisen PS, Petersen RC, Donohue M, Weiner MW, Alzheimer’s Disease Neuroimaging I (2015) Alzheimer’s disease neuroimaging initiative 2 clinical core: progress and plans. Alzheimers Dement 11:734–739. 10.1016/j.jalz.2015.05.005 26194309 10.1016/j.jalz.2015.05.005 PMC4643840

[40] Weiner MW, Veitch DP, Aisen PS et al (2017) The Alzheimer’s disease neuroimaging initiative 3: continued innovation for clinical trial improvement. Alzheimers Dement 13:561–571. 10.1016/j.jalz.2016.10.006 27931796 10.1016/j.jalz.2016.10.006 PMC5536850

[41] Franzmeier N, Neitzel J, Rubinski A et al (2020) Functional brain architecture is associated with the rate of tau accumulation in Alzheimer’s disease. Nat Commun 10.1038/s41467-019-14159-1 10.1038/s41467-019-14159-1 PMC6969065 31953405

[42] Lin Q, Rosenberg MD, Yoo K, Hsu TW, O’Connell TP, Chun MM (2018) Resting-state functional connectivity predicts cognitive impairment related to Alzheimer’s disease. Front Aging Neurosci 10:94. 10.3389/fnagi.2018.00094 29706883 10.3389/fnagi.2018.00094 PMC5908906

[43] Biel D, Luan Y, Brendel M et al (2022) Combining tau-PET and fMRI meta-analyses for patient-centered prediction of cognitive decline in Alzheimer’s disease. Alzheimers Res Ther 14:166. 10.1186/s13195-022-01105-5 36345046 10.1186/s13195-022-01105-5 PMC9639286

[44] Franzmeier N, Caballero MAA, Taylor ANW et al (2017) Resting-state global functional connectivity as a biomarker of cognitive reserve in mild cognitive impairment. Brain Imaging Behav 11:368–382. 10.1007/s11682-016-9599-1 27709513 10.1007/s11682-016-9599-1

[45] Gao YR, Sengupta A, Li MW et al (2020) Functional connectivity of white matter as a biomarker of cognitive decline in Alzheimer’s disease. PLoS One. 10.1371/journal.pone.0240513 10.1371/journal.pone.0240513 PMC7567362 33064765

[46] Xiong Y, Ye CH, Chen Y et al (2022) Altered functional connectivity of basal ganglia in mild cognitive impairment and Alzheimer’s disease. Brain Sci. 10.3390/brainsci12111555 10.3390/brainsci12111555 PMC9688931 36421879

[47] Patel SH, Poisson LM, Brat DJ et al (2017) T2-FLAIR mismatch, an imaging biomarker for IDH and 1p/19q status in lower-grade gliomas: a TCGA/TCIA project. Clin Cancer Res 23:6078–6085. 10.1158/1078-0432.CCR-17-0560 28751449 10.1158/1078-0432.CCR-17-0560

[48] Zhao H, Li W, Lyu P et al (2021) TCGA-TCIA-based CT radiomics study for noninvasively predicting epstein-barr virus status in gastric cancer. AJR Am J Roentgenol 217:124–134. 10.2214/AJR.20.23534 33955777 10.2214/AJR.20.23534

[49] Zanfardino M, Pane K, Mirabelli P, Salvatore M, Franzese M (2019) TCGA-TCIA impact on radiogenomics cancer research: a systematic review. Int J Mol Sci. 10.3390/ijms20236033 10.3390/ijms20236033 PMC6929079 31795520

[50] Yan J, Sun Q, Tan X et al (2023) Image-based deep learning identifies glioblastoma risk groups with genomic and transcriptomic heterogeneity: a multi-center study. Eur Radiol 33:904–914. 10.1007/s00330-022-09066-x 36001125 10.1007/s00330-022-09066-x

[51] Liu D, Chen J, Ge H et al (2023) Radiogenomics to characterize the immune-related prognostic signature associated with biological functions in glioblastoma. Eur Radiol 33:209–220. 10.1007/s00330-022-09012-x 35881182 10.1007/s00330-022-09012-x

[52] Bakas S, Sako C, Akbari H et al (2022) The University of Pennsylvania glioblastoma (UPenn-GBM) cohort: advanced MRI, clinical, genomics, & radiomics. Sci Data 9:453. 10.1038/s41597-022-01560-7 35906241 10.1038/s41597-022-01560-7 PMC9338035

[53] Calabrese E, Villanueva-Meyer JE, Rudie JD et al (2022) The University of California San Francisco preoperative diffuse glioma MRI dataset. Radiol Artif Intell 4:e220058. 10.1148/ryai.220058 36523646 10.1148/ryai.220058 PMC9748624

[54] Des Ligneris M, Bonnet A, Chatelain Y et al (2023) Reproducibility of tumor segmentation outcomes with a deep learning model. International symposium on biomedical imaging (ISBI). Cartagena de Indias, Colombia

[55] Palk A, Illes J, Thompson PM, Stein DJ (2020) Ethical issues in global neuroimaging genetics collaborations. Neuroimage 221:117208. 10.1016/j.neuroimage.2020.117208 32736000 10.1016/j.neuroimage.2020.117208

[56] Bearden CE, Thompson PM (2017) Emerging global initiatives in neurogenetics: the enhancing neuroimaging genetics through meta-analysis (ENIGMA) consortium. Neuron 94:232–236. 10.1016/j.neuron.2017.03.033 28426957 10.1016/j.neuron.2017.03.033 PMC5918136

[57] Schmaal L, Hibar DP, Samann PG et al (2017) Cortical abnormalities in adults and adolescents with major depression based on brain scans from 20 cohorts worldwide in the ENIGMA major depressive disorder working group. Mol Psychiatr 22:900–909. 10.1038/mp.2016.60 10.1038/mp.2016.60 PMC5444023 27137745

[58] Schmaal L, Veltman DJ, van Erp TG et al (2016) Subcortical brain alterations in major depressive disorder: findings from the ENIGMA major depressive disorder working group. Mol Psychiatry 21:806–812. 10.1038/mp.2015.69 26122586 10.1038/mp.2015.69 PMC4879183

[59] van Erp TG, Hibar DP, Rasmussen JM et al (2016) Subcortical brain volume abnormalities in 2028 individuals with schizophrenia and 2540 healthy controls via the ENIGMA consortium. Mol Psychiatry 21:585. 10.1038/mp.2015.118 26283641 10.1038/mp.2015.118 PMC5751698

[60] Boedhoe PSW, Heymans MW, Schmaal L et al (2018) An empirical comparison of meta- and mega-analysis with data from the ENIGMA obsessive–compulsive disorder working group. Front Neuroinform 12:102. 10.3389/fninf.2018.00102 30670959 10.3389/fninf.2018.00102 PMC6331928

[61] Bruin WB, Abe Y, Alonso P et al (2023) The functional connectome in obsessive-compulsive disorder: resting-state mega-analysis and machine learning classification for the ENIGMA-OCD consortium. Mol Psychiatry. 10.1038/s41380-023-02077-0. 10.1038/s41380-023-02077-0 PMC10827654 37131072

[62] Liew SL, Zavaliangos-Petropulu A, Jahanshad N et al (2022) The ENIGMA stroke recovery working group: big data neuroimaging to study brain-behavior relationships after stroke. Human Brain Mapping 43:129–148. 10.1002/hbm.25015 32310331 10.1002/hbm.25015 PMC8675421

[63] Cheon EJ, Bearden CE, Sun D et al (2022) Cross disorder comparisons of brain structure in schizophrenia, bipolar disorder, major depressive disorder, and 22q11.2 deletion syndrome: aA review of ENIGMA findings. Psychiatry Clin Neurosci 76:140–161. 10.1111/pcn.13337 35119167 10.1111/pcn.13337 PMC9098675

[64] Thompson PM, Stein JL, Medland SE et al (2014) The ENIGMA consortium: large-scale collaborative analyses of neuroimaging and genetic data. Brain Imaging Behav 8:153–182. 10.1007/s11682-013-9269-5 24399358 10.1007/s11682-013-9269-5 PMC4008818

[65] Zugman A, Harrewijn A, Cardinale EM et al (2022) Mega-analysis methods in ENIGMA: the experience of the generalized anxiety disorder working group. Hum Brain Mapp 43:255–277. 10.1002/hbm.25096 32596977 10.1002/hbm.25096 PMC8675407

[66] Thompson PM, Jahanshad N, Schmaal L et al (2022) The enhancing neuroimaging genetics through meta-analysis consortium: 10 years of global collaborations in human brain mapping. Human Brain Mapping 43:15–22. 10.1002/hbm.25672 34612558 10.1002/hbm.25672 PMC8675422

[67] Koile E, Cristia A (2021) Toward cumulative cognitive science: a comparison of meta-analysis, mega-analysis, and hybrid approaches. Open Mind 5:154–173. 10.1162/opmi_a_00048 35024529 10.1162/opmi_a_00048 PMC8746126

[68] Amunts K, Ebell C, Muller J, Telefont M, Knoll A, Lippert T (2016) The human brain project: creating a european research infrastructure to decode the human brain. Neuron 92:574–581. 10.1016/j.neuron.2016.10.046 27809997 10.1016/j.neuron.2016.10.046

[69] Marcus DS, Wang TH, Parker J, Csernansky JG, Morris JC, Buckner RL (2007) Open access series of imaging studies (OASIS): cross-sectional MRI data in young, middle aged, nondemented, and demented older adults. J Cogn Neurosci 19:1498–1507. 10.1162/jocn.2007.19.9.1498 17714011 10.1162/jocn.2007.19.9.1498

[70] Shafto MA, Tyler LK, Dixon M et al (2014) The Cambridge Centre for Ageing and Neuroscience (Cam-CAN) study protocol: a cross-sectional, lifespan, multidisciplinary examination of healthy cognitive ageing. BMC Neurol 14:204. 10.1186/s12883-014-0204-1 25412575 10.1186/s12883-014-0204-1 PMC4219118

[71] Taylor JR, Williams N, Cusack R et al (2017) The Cambridge Centre for Ageing and Neuroscience (Cam-CAN) data repository: structural and functional MRI, MEG, and cognitive data from a cross-sectional adult lifespan sample. Neuroimage 144:262–269. 10.1016/j.neuroimage.2015.09.018 26375206 10.1016/j.neuroimage.2015.09.018 PMC5182075

[72] Holmes AJ, Hollinshead MO, O’Keefe TM et al (2015) Brain genomics superstruct project initial data release with structural, functional, and behavioral measures. Sci Data 2:150031. 10.1038/sdata.2015.31 26175908 10.1038/sdata.2015.31 PMC4493828

[73] Parkinson Progression Marker I (2011) The Parkinson progression marker initiative (PPMI). Prog Neurobiol 95:629–635. 10.1016/j.pneurobio.2011.09.005 21930184 10.1016/j.pneurobio.2011.09.005 PMC9014725

[74] Di Martino A, Yan CG, Li Q et al (2014) The autism brain imaging data exchange: towards a large-scale evaluation of the intrinsic brain architecture in autism. Mol Psychiatry 19:659–667. 10.1038/mp.2013.78 23774715 10.1038/mp.2013.78 PMC4162310

[75] Consortium HD. (2012) The ADHD-200 consortium: a model to advance the translational potential of neuroimaging in clinical neuroscience. Front Syst Neurosci 6:62. 10.3389/fnsys.2012.00062 22973200 10.3389/fnsys.2012.00062 PMC3433679

[76] Horien C, Lee K, Westwater ML et al (2022) A protocol for working with open-source neuroimaging datasets. STAR Protoc 3:101077. 10.1016/j.xpro.2021.101077 35036958 10.1016/j.xpro.2021.101077 PMC8749295

[77] Dvir Dov RT, Shenhar Aaron (2003) An empirical analysis of the relationship between project planning and project success. Int J Proj Manag 21:89–95. 10.1016/S0263-7863(02)00012-1

[78] Stroganov O, Fedarovich A, Wong E et al (2022) Mapping of UK Biobank clinical codes: challenges and possible solutions. PLoS One. 10.1371/journal.pone.0275816 10.1371/journal.pone.0275816 PMC9757572 36525430

[79] Eshaghzadeh Torbati M, Minhas DS, Ahmad G et al (2021) A multi-scanner neuroimaging data harmonization using RAVEL and ComBat. Neuroimage 245:118703. 10.1016/j.neuroimage.2021.118703 34736996 10.1016/j.neuroimage.2021.118703 PMC8820090

[80] Gorgolewski KJ, Auer T, Calhoun VD et al (2016) The brain imaging data structure, a format for organizing and describing outputs of neuroimaging experiments. Sci Data 3:160044. 10.1038/sdata.2016.44 27326542 10.1038/sdata.2016.44 PMC4978148

[81] Niso G, Botvinik-Nezer R, Appelhoff S et al (2022) Open and reproducible neuroimaging: from study inception to publication. Neuroimage 263:119623. 10.1016/j.neuroimage.2022.119623 36100172 10.1016/j.neuroimage.2022.119623 PMC10008521

[82] Fezai L, Urruty T, Bourdon P, Fernandez-Maloigne C, Initi AsDN (2023) Deep anonymization of medical imaging. Multimed Tools Appl 82:9533–9547. 10.1007/s11042-022-13686-2

[83] Nichols TE, Das S, Eickhoff SB et al (2017) Best practices in data analysis and sharing in neuroimaging using MRI. Nat Neurosci 20:299–303. 10.1038/nn.4500 28230846 10.1038/nn.4500 PMC5685169

[84] Aerts H, Schirner M, Jeurissen B et al (2018) Modeling brain dynamics in brain tumor patients using the virtual brain. eNeuro. 10.1523/ENEURO.0083-18.2018 10.1523/ENEURO.0083-18.2018 PMC6001263 29911173

[85] Aerts H, Schirner M, Dhollander T et al (2020) Modeling brain dynamics after tumor resection using the virtual brain. Neuroimage 213:116738. 10.1016/j.neuroimage.2020.116738 32194282 10.1016/j.neuroimage.2020.116738

[86] Menze BH, Jakab A, Bauer S et al (2015) The multimodal brain tumor image segmentation benchmark (BRATS). IEEE T Med Imaging 34:1993–2024. 10.1109/Tmi.2014.2377694 10.1109/TMI.2014.2377694 PMC4833122 25494501

[87] Bakas S, Akbari H, Sotiras A et al (2017) Data descriptor: advancing the cancer genome atlas glioma MRI collections with expert segmentation labels and radiomic features. Scientific Data. 10.1038/sdata.2017.117 10.1038/sdata.2017.117 PMC5685212 28872634

[88] Jwa AS, Poldrack RA (2022) The spectrum of data sharing policies in neuroimaging data repositories. Human Brain Mapping 43:2707–2721. 10.1002/hbm.25803 35142409 10.1002/hbm.25803 PMC9057092

[89] Jenkinson M, Beckmann CF, Behrens TE, Woolrich MW, Smith SM (2012) FSL. Neuroimage 62:782–790. 10.1016/j.neuroimage.2011.09.015 21979382 10.1016/j.neuroimage.2011.09.015

[90] Frackowiak RS (2004) Human brain function. Elsevier, Amsterdam

[91] Cox RW (1996) AFNI: software for analysis and visualization of functional magnetic resonance neuroimages. Comput Biomed Res 29:162–173. 10.1006/cbmr.1996.0014 8812068 10.1006/cbmr.1996.0014

[92] Avants BB, Tustison NJ, Song G, Cook PA, Klein A, Gee JC (2011) A reproducible evaluation of ANTs similarity metric performance in brain image registration. Neuroimage 54:2033–2044. 10.1016/j.neuroimage.2010.09.025 20851191 10.1016/j.neuroimage.2010.09.025 PMC3065962

[93] Fischl B (2012) FreeSurfer. Neuroimage 62:774–781. 10.1016/j.neuroimage.2012.01.021 22248573 10.1016/j.neuroimage.2012.01.021 PMC3685476

[94] Esteban O, Markiewicz CJ, Blair RW et al (2019) fMRIPrep: a robust preprocessing pipeline for functional MRI. Nat Methods 16:111–116. 10.1038/s41592-018-0235-4 30532080 10.1038/s41592-018-0235-4 PMC6319393

[95] Sikka S, Cheung B, Khanuja R et al (2014) Towards automated analysis of connectomes: the configurable pipeline for the analysis of connectomes (c-pac). In: 5th INCF Congress of Neuroinformatics, Munich

[96] Li X, Bianchini Esper N, Ai L et al (2024) Moving beyond processing- and analysis-related variation in resting-state functional brain imaging. Nat Hum Behav 8:2003–2017. 10.1038/s41562-024-01942-4 10.1038/s41562-024-01942-4 39103610

[97] Gorgolewski K, Burns CD, Madison C et al (2011) Nipype: a flexible, lightweight and extensible neuroimaging data processing framework in python. Front Neuroinform 5:13. 10.3389/fninf.2011.00013 21897815 10.3389/fninf.2011.00013 PMC3159964

[98] Abraham A, Pedregosa F, Eickenberg M et al (2014) Machine learning for neuroimaging with scikit-learn. Front Neuroinform 8:14. 10.3389/fninf.2014.00014 24600388 10.3389/fninf.2014.00014 PMC3930868

[99] Bowring A, Maumet C, Nichols TE (2019) Exploring the impact of analysis software on task fMRI results. Hum Brain Mapp 40:3362–3384. 10.1002/hbm.24603 31050106 10.1002/hbm.24603 PMC6618324

[100] Luppi AI, Gellersen HM, Liu ZQ et al (2024) Systematic evaluation of fMRI data-processing pipelines for consistent functional connectomics. Nat Commun 15:4745. 10.1038/s41467-024-48781-5 38834553 10.1038/s41467-024-48781-5 PMC11150439

[101] Desmond JE, Glover GH (2002) Estimating sample size in functional MRI (fMRI) neuroimaging studies: statistical power analyses. J Neurosci Methods 118:115–128. 10.1016/s0165-0270(02)00121-8 12204303 10.1016/s0165-0270(02)00121-8

[102] Szucs D, Ioannidis JP (2020) Sample size evolution in neuroimaging research: an evaluation of highly-cited studies (1990-2012) and of latest practices (2017–2018) in high-impact journals. Neuroimage 221:117164. 10.1016/j.neuroimage.2020.117164 32679253 10.1016/j.neuroimage.2020.117164

[103] Marek S, Tervo-Clemmens B, Calabro FJ et al (2022) Reproducible brain-wide association studies require thousands of individuals. Nature 603:654–660. 10.1038/s41586-022-04492-9 35296861 10.1038/s41586-022-04492-9 PMC8991999

[104] Goddard M (2017) The EU general data protection regulation (GDPR): European regulation that has a global impact. Int J Market Res 59:703–705. 10.2501/Ijmr-2017-050

[105] Rosenbaum S (2010) Data governance and stewardship: designing data stewardship entities and advancing data access. Health Serv Res 45:1442–1455. 10.1111/j.1475-6773.2010.01140.x 21054365 10.1111/j.1475-6773.2010.01140.x PMC2965885

[106] Jin H, Luo Y, Li P, Mathew J (2019) A review of secure and privacy-preserving medical data sharing. IEEE Access 7:61656–61669

[107] Hudson KL, Collins FS (2015) Bringing the common rule into the 21st century. New Engl J Med 373:2293–2296. 10.1056/NEJMp1512205 26509903 10.1056/NEJMp1512205 PMC5101946

[108] Mulgund P, Mulgund BP, Sharman R, Singh R (2021) The implications of the California Consumer Privacy Act (CCPA) on healthcare organizations: lessons learned from early compliance experiences. Health Policy Technol. 10.1016/j.hlpt.2021.100543

[109] United S (1996) Health insurance portability and accountability act of 1996. Public law 104–191. US Statut Large 110:1936–2103 16477734

[110] Sarabdeen J, Chikhaoui E, Mohamed Ishak MM (2022) Creating standards for Canadian health data protection during health emergency—an analysis of privacy regulations and laws. Heliyon 8:e09458. 10.1016/j.heliyon.2022.e09458 35637667 10.1016/j.heliyon.2022.e09458 PMC9142849

[111] Shen FX, Wolf SM, Lawrenz F et al (2024) Ethical, legal, and policy challenges in field-based neuroimaging research using emerging portable MRI technologies: guidance for investigators and for oversight. J Law Biosci 11:lsae008. 10.1093/jlb/lsae008 38855036 10.1093/jlb/lsae008 PMC11157461

[112] Hamet P, Tremblay J (2017) Artificial intelligence in medicine. Metabolism 69S:S36–S40. 10.1016/j.metabol.2017.01.011 28126242 10.1016/j.metabol.2017.01.011

[113] Holzinger A, Langs G, Denk H, Zatloukal K, Muller H (2019) Causability and explainability of artificial intelligence in medicine. Wiley Interdiscip Rev Data Min Knowl Discov 9:e1312. 10.1002/widm.1312 32089788 10.1002/widm.1312 PMC7017860

[114] Reyes M, Meier R, Pereira S et al (2020) On the interpretability of artificial intelligence in radiology: challenges and opportunities. Radiol Artif Intell 2:e190043. 10.1148/ryai.2020190043 32510054 10.1148/ryai.2020190043 PMC7259808

[115] Ienca M, Ignatiadis K (2020) Artificial intelligence in clinical neuroscience: methodological and ethical challenges. AJOB Neurosci 11:77–87. 10.1080/21507740.2020.1740352 32228387 10.1080/21507740.2020.1740352

[116] Liebrenz M, Schleifer R, Buadze A, Bhugra D, Smith A (2023) Generating scholarly content with ChatGPT: ethical challenges for medical publishing. Lancet Digit Health 5:e105–e106. 10.1016/S2589-7500(23)00019-5 36754725 10.1016/S2589-7500(23)00019-5

[117] Challen R, Denny J, Pitt M, Gompels L, Edwards T, Tsaneva-Atanasova K (2019) Artificial intelligence, bias and clinical safety. BMJ Qual Saf 28:231–237. 10.1136/bmjqs-2018-008370 30636200 10.1136/bmjqs-2018-008370 PMC6560460

[118] Parikh RB, Teeple S, Navathe AS (2019) Addressing bias in artificial intelligence in health care. JAMA 322:2377–2378. 10.1001/jama.2019.18058 31755905 10.1001/jama.2019.18058

[119] Boochever SS (2004) HIS/RIS/PACS integration: getting to the gold standard. Radiol Manage 26:16–24. quiz 5-7 15259683

[120] Rao TS, Radhakrishnan R, Andrade C (2011) Standard operating procedures for clinical practice. Indian J Psychiatry 53:1–3. 10.4103/0019-5545.75542 21430999 10.4103/0019-5545.75542 PMC3056180

[121] Simmons JP, Nelson LD, Simonsohn U (2021) Pre-registration: why and how. J Consum Psychol 31:151–162. 10.1002/jcpy.1208

[122] Van ‘t Veer AE, Giner-Sorolla R (2016) Pre-registration in social psychology—a discussion and suggested template. J Exp Soc Psychol 67:2–12. 10.1016/j.jesp.2016.03.004

[123] Logg JM, Dorison CA (2021) Pre-registration: weighing costs and benefits for researchers. Organ Behav Hum 167:18–27. 10.1016/j.obhdp.2021.05.006

[124] Ross MH (1993) The management of conflict: Interpretations and interests in comparative perspective. Yale University Press, New Haven

[125] Roche WK, Teague P, Colvin AJ (2014) The Oxford handbook of conflict management in organizations. Oxford University Press, New York

